# In vitro anti‐*Trichomonas gallinae* effects of *Ziziphus vulgaris* L. and *Camellia sinensis* (L.) Kuntze extracts

**DOI:** 10.1002/vms3.1432

**Published:** 2024-03-25

**Authors:** Behnam Rahimi, Farnaz Malekifard, Bijan Esmaeilnejad

**Affiliations:** ^1^ DVM graduate Faculty of Veterinary Medicine Urmia University Urmia Iran; ^2^ Department of Pathobiology Faculty of Veterinary Medicine Urmia University Urmia Iran

**Keywords:** *Camellia sinensis* (L.) Kuntze, in vitro, *Trichomonas gallinae*, *Ziziphus vulgaris* L. (jujube)

## Abstract

**Background:**

*Trichomonas gallinae* is a parasite that causes canker and severe loss and death, especially in young pigeons. Metronidazole (MTZ) is the recommended drug for treating avian trichomoniasis. Due to drug resistance, non‐chemical alternatives, such as medicinal plant extracts, are also considered possible therapies for this disease.

**Objectives:**

This study compares the antitrichomonal effects of MTZ with extracts of *Camellia sinensis* and *Ziziphus vulgaris* on *T. gallinae* in vitro.

**Methods:**

Samples of *T. gallinae* were taken from infected pigeons. Multi‐well plates with different concentrations (5, 10, 25, 50 and 100 µg/mL) of plant extracts were used for the in vitro study.

**Results:**

The minimum inhibitory concentration (MIC) of *C. sinensis* extract was 25 µg/mL over 24 h, compared to 50 µg/mL for MTZ. The MIC value of the *Z. vulgaris* extracts was 50 µg/mL.

**Conclusions:**

The results suggest that the extracts of *Z. vulgaris* and *C. sinensis*, as potential natural agents, could have anti‐avian trichomoniasis properties. This study also shows that MTZ, *C. sinensis* and *Z. vulgaris* are equally effective in preventing the growth of *T. gallinae* trophozoites in the culture.

## Graphical abstract

Graphical Abstract: *Ziziphus vulgaris* L. (jujube) and *Camellia sinensis* (L.) Kuntze extracts have anti‐*Trichomonas gallinae* effects and could be used as potent natural anti‐*Trichomonas gallinae* agents. In addition, metronidazole, *C. sinensis* and *Z. vulgaris* extracts equally prevent the growth of *T. gallinae* trophozoites in the culture.

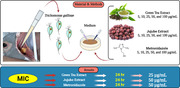

## 1 INTRODUCTION

*Trichomonas gallinae*, a flagellated protozoon that causes trichomoniasis in birds, belongs to the order Trichomonadida. This parasite infects the upper gastrointestinal tract of a variety of birds, including Columbiformes (doves and domestic pigeons) (Boal et al., 1998; Forrester & Foster, 2008; Rouffaer et al., 2014). A common symptom of the condition is caseous ulcers in the front part of the digestive system. The severity of the lesions can vary, and in certain situations they can kill birds by obstructing the oesophageal lumen (Gerhold et al., 2008).

Pigeons with avian trichomoniasis are treated with various medications. Metronidazole (MTZ) is the drug of choice for avian trichomoniasis (Aydın et al., 2000; Seddiek et al., 2014) and should be used with caution due to the risk of carcinogenicity and drug resistance (Upcroft & Upcroft, 2001). MTZ has various side effects, including transient neutropenia, nausea, cancer and peripheral neuropathy (Kurohara et al., 1991; Sobel et al., 1999). Scientists are looking for non‐chemical and natural alternatives to chemical drugs to get rid of parasites due to the side effects of chemical drugs (Khater, 2012, 2013, 2011; Seddiek et al., 2014).

Green tea, the dried leaf of the *Camellia sinensis* plant, contains a number of physiologically active substances, including polyphenols, methylxanthines, essential oils, proteins, vitamins and amino acids (Yamamoto et al., 1997). Previous research has focused on the extraction of *C. sinensis* polyphenols (Perva‐Uzunalić et al., 2006; Senanayake, 2013) and testing of *C. sinensis* extracts for potential antioxidant, antiviral and antitumor properties (Adhami & Mukhtar, 2007; Dai & Mumper, 2010; Feitelson et al., 2015; Yang et al., 2019). The most common ingredient in *C. sinensis* is catechins (Benelli et al., 2002). Tea catechins are primarily responsible for the biological benefits of *C. sinensis*, including lowering plasma lipid levels, reducing inflammation and having antibacterial, antiparasitic, anticancer and antioxidant properties (Bohm, 1998; Huang et al., 1998; Paveto et al., 2004; Vijaya et al., 1995). These chemicals, which belong to the flavan‐3‐ol family, have attracted a lot of interest lately due to their potential therapeutic properties. Among other health benefits, the powerful antioxidant and antiviral properties of catechins could help prevent disease (Katada et al., 2020; Sanlier et al., 2018; Yang et al., 2019). According to the study by Paveto et al. (2004) on the anti‐*Trypanosoma cruzi* activity of green tea in vitro, green tea catechins are effective therapeutics for Chagas disease.

*Ziziphus vulgaris* (jujube) belongs to the plant family Rhamnaceae. This plant is widely cultivated in Burma, Iran and several regions of India (Goli‐malekabadi et al., 2014). According to phytochemical analysis, *Z. vulgaris* has been shown to contain bioactive substances, including cyclopeptide alkaloids. According to several studies, cyclopeptide alkaloids are the cause of the antiplasmodial and antimycobacterial abilities of *Ziziphus*. In addition, the plant has been found to contain terpenoids, alkaloids, steroids, polysaccharides and saponin glycosides (Dodangeh et al., 2017; Panseeta et al., 2011). Dodangeh et al. (2017) showed that *Z. vulgaris* extract induces programmed death in *Acanthamoeba* cysts and trophozoites in culture. Jujube is therefore considered a potential alternative medicinal plant for new antiparasitic drugs.

To our knowledge, no one has taken note of the antitrichomonal properties of *C. sinensis* and *Z. vulgaris*. So, the aim of this work is to compare the in vitro antitrichomonal effects of *C. sinensis* and *Z. vulgaris* against *T. gallinae* with those of MTZ.

## 2 MATERIALS AND METHODS

### 2.1 Preparation of plant extract

The *Z. vulgaris* and *C. sinensis* plants were purchased from a Persian herbal market and confirmed by the Agriculture Faculty of Urmia University, Iran. The technique utilized by Baqer et al. (2014) underwent certain adjustments, and plant extracts were prepared. All the dry plant materials, leaves of green tea and dried fruits of jujube were put in an electrical blender (Moulinex) and turned into powder. Five hundred mL of 70% ethanol and 100 g of the powdered plant were combined with a magnetic stirrer and stirred for 2 h. The obtained solution was left intact at room temperature for 24 h and was filtered after stirring again. The solvent was removed using a rotary evaporator. For future applications, the semi‐solid residual components were freeze‐dried at 4°C.

### 2.2 Gas chromatography–mass spectrometry (GC–MS) analysis

GC–MS (Thermo Scientific) was used to examine the chemical composition of the extract. The split ratio for the helium‐based carrier gas was 0.50 mL/min. The following GS conditions were met: initial 40°C ramping to 250°C, 80°C per min for 3 min, with 250°C injector and detector temperatures. Individual chemicals were identified by comparing their relative retention times on a capillary column to those of real samples and their peak‐to‐peak mass spectra to those obtained from authentic samples and published data (Khoshnejad et al., 2023).

### 2.3 Parasites

Twenty‐five pigeons were purchased from a local breeder in Urmia, Iran. The pigeons were about 6‐week old. *T. gallinae* was recovered using the wet mounting technique. Microbiological swabs moistened with the warm saline solution were used to collect samples from membrane lesions in the oropharyngeal region of the birds. Wet smears were prepared by rubbing the swabs on the glass slide. The approach of Samour and Naldo (2003) was used to confirm *T. gallinae* on the slides when magnified with a light microscope at 100× and 400× magnification. Flagellated and pear‐shaped trophozoites could be seen under the microscope. *T. gallinae* trophozoites were extracted from the mouth (Seddiek et al., 2014). Diamond's trypticase, yeast extract and maltose (TYM) medium (Oxoid Ltd.) was used to prepare the parasite cultures (Tabari et al., 2017). The culture received 10% foetal calf serum (Sigma Chemical Co.) and was incubated at 37°C. After 5 days, the cultures were examined to assess the growth of *T. gallinae* (Seddiek et al., 2014). Penicillin and streptomycin (120 IU) were added in the initial phases to the subcultures; but antibiotic use was discontinued once axenic culture was achieved (Tabari et al., 2017).

### 2.4 In vitro assay

We performed the in vitro analysis using the Tabari et al. (2017) approach with some modifications. MTZ (Alborz Daru) was used as a conventional anti‐*T. gallinae* drug. Dimethylsulfoxide (DMSO, 0.5%) was used to dilute MTZ and all plant extracts (Bharti et al., 2006; Malekifard et al., 2021). The response of *T. gallinae* to these substances was investigated by incubating the trophozoites with different concentrations of *Z. vulgaris*, *C. sinensis* extracts and MTZ in multi‐well plates. One hundred microliters of culture medium were added to each well. Prediluted MTZ, *Z. vulgaris* and *C. sinensis* extracts, and 1 × 10^4^ parasites were added to the culture to achieve final concentrations of 5, 10, 25, 50 and 100 µg/mL. The control was the plate with no treatment. The wells were sealed by spreading Vaseline (50 µL) over them to create an anaerobic environment. The plates were then incubated for a further 72 h at a temperature of 37°C (Tabari et al., 2017). Cultured trophozoites were counted with a hemocytometer. Trypan blue (0.4%) was added to the samples in equal parts to help distinguish between live and dead trophozoites (Tabari et al., 2017). At least three replicates of each experiment were used to obtain the results. The lowest concentration of a drug at which the parasites were no longer motile was called the minimum inhibitory concentration (MIC) (Seddiek et al., 2014). The following equation was used to calculate the growth inhibition (GI) percentage:

$$\mathrm{Growth}\mathrm{inhibition}\left(\%\right)=\left(A-B\right)/A\times 100$$

In the above equation, *A* denotes the average number of trophozoites in the control group and *B* denotes the average number of trophozoites in the test group (Seddiek et al., 2014).

### 2.5 Statistical analysis

Statistical analysis was performed by SPSS (v. 26.0). An analysis of variance examined the difference between the control and test groups. Values below 0.05 (*p *< 0.05) were considered significant.

## 3 RESULTS

### 3.1 Gas chromatography–mass spectrometry analysis

Based on GC/MS analysis of the studied extracts, catechin (22.67%), quercitrin (19.83%), Kaempferol (14.44%), quinic acid (11.45%) in *Z. vulgaris* extract (Table 1), caffeine (24.07%), naphthacene (12.36%), squalene (11.34%) and *N*‐hexadecanoic acid (9.32%) in *C. sinensis* extract (Table 1) were the most important chemical components.

**TABLE 1:** Major chemical compounds in *Ziziphus vulgaris and C*amellia *sinensis* extract identified by gas chromatography–mass spectrometry (GC–MS).

Plant extract	Major compounds	Percent
*Z. vulgaris*	Quinic acid	11.45
	Gallic acid	8.74
	Catechin	22.67
	Epicatechin	2.08
	Quercitrin	19.83
	Naringin	5.34
	Acacetin	2.13
	Kaempferol	14.44
	3,4‐di‐*O*‐caffeoylquinic acid	1.22
	1,3‐di‐*O*‐caffeoylquinic acid	4.56
*C. sinensis*	Squalene	11.34
	Methyl stearate	3.05
	Methyl linoleate	7.43
	Caffeine	24.07
	Naphthacene	12.36
	α‐Bulnesene	2.52
	Eremophilene	1.22
	γ‐Muurolene	1.38
	*N*‐hexadecanoic acid	9.32
	Methyl hexadecanoate	0.43

### 3.2 In vitro results

Efficacy data for various concentrations of *Z. vulgaris* and *C. sinensis* extracts as anti‐*T. gallinae* agents are presented in Table 2. It could be observed that the extracts of *Z. vulgaris* and *C. sinensis* had antitrichomonal activity against *T. gallinae* in different concentrations and times. *C. sinensis* extract had a MIC of 25 µg/mL at 24 h compared to 50 µg/mL for MTZ. For *Z. vulgaris*, these doses were 50 and 10 µg/mL in 48 and 72 h, respectively. It is interesting to note that the MIC for MTZ was determined to be 25 and 10 µg/mL at 48 and 72 h, respectively. According to the study, *C. sinensis* was able to completely eliminate *T. gallinae* in 48 h at the MIC of 25 µg/mL but took 72 h at the MIC of 10 µg/mL (Table 2). The control samples showed no decrease in cell populations.

**TABLE 2:** Effect of various metronidazole, *Ziziphus vulgaris* and *Camellia sinensis* extract concentrations on the in vitro growth of *Trichomonas gallinae* trophozoites (10^4^).

Treatment	Concentration (µg/mL)	Trophozoites number		
		24 h	48 h	72 h
*C. sinensis*	5	6.3 ± 0.33^b^	1.46 ± 0.51^b^	0.67 ± 0.32^b^
	10	2.06 ± 0.12^c^	0.47 ± 0.17 ^c^	0^b^
	25	0^d^	0^c^	0^b^
	50	0^d^	0^c^	0^b^
	100	0^d^	0^c^	0^b^
*Z. vulgaris*	5	5.61 ± 0.08^b^	1.45 ± 0.37^b^	0.65 ± 0.13^b^
	10	1.43 ± 0.34^c^	0.65 ± 0.20^c^	0^b^
	25	0.55 ± 0.12^d^	0.06 ± 0.45^c^	0^b^
	50	0^d^	0^c^	0^b^
	100	0^d^	0^c^	0^b^
Metronidazole	5	5.32 ± 0.44^b^	2.13 ± 0.57^b^	0.78 ± 0.17^b^
	10	2.02 ± 0.67^c^	0.56 ± 0.04^c^	0^b^
	25	0.42 ± 0.06^d^	0^c^	0^b^
	50	0^d^	0^c^	0^b^
	100	0^d^	0^c^	0^b^
Control	–	8.54 ± 0.22^a^	7.68 ± 0.05^a^	6.75 ± 0.35^a^

*Note*: Data are presented as mean ± SD. a‐d Different superscript letters in a column indicate significant differences (*p *< 0.05).

**FIGURE 1:**
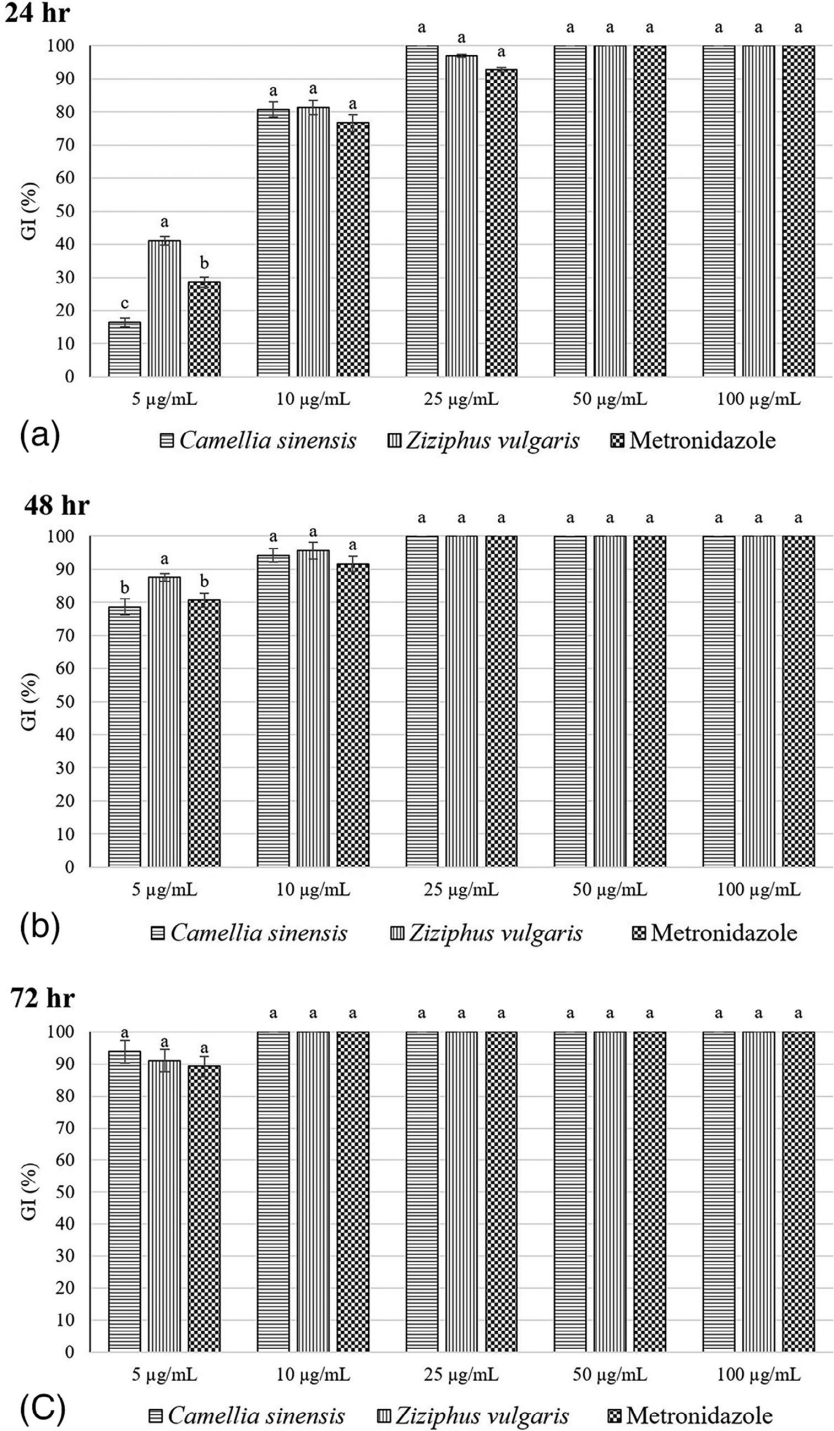
Comparison of growth inhibition percentage (GI%) of *Trichomonas gallinae* trophozoites in the presence of different concentrations of *C*amellia *sinensis, Ziziphus vulgar is* extracts and metronidazole for 24 (A), 48 (B) and 72 (C) h. Different superscript letters in a column indicate significant differences (*p *< 0.05).

Figure 1 shows the per cent GI% results for the groups treated with *Z. vulgaris* and *C. sinensis* extracts and MTZ for 24, 48 and 72 h. The data reveals a significant difference in GI% between the groups treated with *Z. vulgaris* and *C. sinensis* extracts and MTZ as compared to the control group. According to Figure 1, the GI% of *Z. vulgaris* and *C. sinensis* extracts at 10, 25, 50 and 100 µg/mL doses had similar effects to MTZ after 24, 48 and 72 h. In addition, after 72 h, the GI% of these extracts in all doses used was also similar to that of MTZ. Therefore, the study shows that *Z. vulgaris* and *C. sinensis* extracts have a potent anti‐*T. gallinae* effect in vitro.

## 4 DISCUSSION

Recently, there has been a growing interest in using antiparasitic natural extracts to treat various medical conditions. Some herbal extracts play an important role as an alternative to medicines. Several studies have demonstrated the effectiveness of different herbal extracts in eradicating protozoa, such as *Acanthamoeba* spp. (Dodangeh et al., 2017), *Plasmodium falciparum* (Milhau et al., 1997), *Trichomonas vaginalis* (Kaneda et al., 1990, 1991; Zemek et al., 1987),*Trypanosoma brucei* (Moideen et al., 1999),*Cryptosporidium* spp. (Sréter et al., 1999), *Entamoeba histolytica* (Kaneda et al., 1990, 1991)and *Giardia* spp. (Kaneda et al., 1990, 1991). In recent years, the antiplasmodial, antiamoebic, anticoccidial and anti‐*T. cruzi* activity of *C. sinensis* and *Z. vulgaris* extracts were shown in many studies (Aboulaila et al., 2010; Dodangeh et al., 2017; Panseeta et al., 2011; Paveto et al., 2004). In this study, the anti‐*T. gallinae* activity of green tea (*C. sinensis*) and jujube (*Z. vulgaris*) extracts was examined in vitro. To our knowledge, this is the first study of the anti‐*T. gallinae* properties of these extracts.

This study demonstrated the effectiveness of *Z. vulgaris* and *C. sinensis* extracts against *T. gallinae*. The results of our study agree with those of Malekifard et al. (2021), who found anti‐*T. gallinae* effects of *Zingiber officinale*‏‏ and *Lavandula angustifolia* alcoholic extracts, which were dose‐ and time‐dependent. Another study by Tabari et al. demonstrated the antitrichomonal effect of *Peganum harmala* on *T. gallinae*. The MIC for *P. harmala* extract at 24 h was 15 µg/mL compared to 50 µg/mL for MTZ. Their research showed that MTZ‐resistant *T. gallinae* isolates can be successfully treated with the alkaloid extract of *P. harmala* (Tabari et al., 2017).

Our research serves as evidence of the in vitro anti‐*T. gallinae* activity of jujube extract. In this study, we identified catechin (22.67%) in the jujube extract. The jujube extract was found to contain small amounts of other compounds. The results obtained by earlier studies showed the antiparasitic qualities of catechin, quercitrin and Kaempferol, which were the main components of jujube in this study (Calzada, 2005; Dodson et al., 2011; Paveto et al., 2004). In addition, the results of this study support the results of previous investigations into the antiparasitic, antifungal and antimicrobial properties of jujube cyclopeptide alkaloids, tannins and saponins (Chung et al., 1998; Dodangeh et al., 2017; Panseeta et al., 2011). Our in vitro study showed that after adding the jujube extract (50 µg/mL) to the growth medium, trichomonads were no longer present after 24 h. Other studies have shown that jujube extract is effective in vitro against *Acanthamoeba* spp. and malaria (Panseeta et al., 2011). The amoebicidal effect of *Z. vulgaris* is related to its propensity to induce apoptosis, as Dodangeh et al. reported. Previous research showed that inducing apoptosis in *Z. vulgaris* has a significant advantage over other widely used drugs, such as MTZ. The fact that *Z. vulgaris* has no cytotoxic effect on cultured macrophage cells, which is another benefit of the herb (Dodangeh et al., 2017).

In this study, caffeine (24.07%) was the main component of green tea extract, and the antiparasitic effect of this substance was reported in several studies. The results of these studies suggested that green tea extract and caffeine had anti‐*Acanthamoeba* and antileishmanial effects (Hajihossein et al., 2020; Tadesse et al., 2015). According to previous research (Parvez et al., 2019), *C. sinensis* and its components showed antimicrobial activity against gram‐positive and gram‐negative multidrug‐resistant bacteria. In addition, *C. sinensis* inhibited *Leishmania amazonensi*s (dos Reis et al., 2013; Inacio et al., 2013), reduced exposure to *Haemonchus contortus* worms (Zhong et al., 2014), and inhibited promastigote and amastigote forms of *Leishmania braziliensis* (Inacio et al., 2014). In addition, *Babesia* spp., *Eimeria* spp. and *T. cruzi* were all inhibited by *C. sinensis* (Aboulaila et al., 2010; Jang et al., 2007; Paveto et al., 2004). The results of the study by Fakae et al. showed that beers made from *C. sinensis* exhibit amoebic activity against *Acanthamoeba* trophozoites and were very successful in preventing the parasite from encysting. According to their research, *C. sinensis* could be a source of inhibitors of *Acanthamoeba castellanii* growth and encystation, which could be useful in the development of topical adjunctive treatments to supplement the chemotherapeutic drugs currently used to treat *A. castellanii* infections (Fakae et al., 2020).

Avian trichomoniasis is commonly treated with MTZ; however, certain isolates of *T. gallinae* are resistant to the drugs, posing a risk to free‐living birds (Tabari et al., 2017). These isolates have been found in a number of countries, including Spain, Belgium, the United States and Iran (Gerhold et al., 2008; Munoz et al., 1998; Rouffaer et al., 2014; Tabari et al., 2017). According to our research, *Z. vulgaris* and *C. sinensis* extracts can be more effective treatment options for the above isolates. To assess MTZ resistance in *T. gallinae* strains (Rouffaer et al., 2014), a 24‐h MIC breakpoint (15.6 µg/mL) was established. Our study revealed that the 24 h MIC of MTZ was 50 µg/mL, which differs significantly from that reported by Rouffaer et al., whose specified value differs. As a result, we were able to conclude that the *T. gallinae* strains used in this study exhibited high levels of MTZ resistance (Rouffaer et al., 2014). Our results agreed with those of Tabari et al. (2017) and Malekifard et al. (2021), who confirmed the presence of MTZ‐resistant strains of *T. gallinae* in Iran.

## 5 CONCLUSIONS

The results of this study suggest that *Z. vulgaris* and *C. sinensis* extracts could be used as potent natural anti*‐T. gallinae* agents. Further research is needed to determine the potential adverse effects of *Z. vulgaris* and *C. sinensis* and to demonstrate their  trichomonicidal properties in vivo.

## AUTHOR CONTRIBUTIONS

Farnaz Malekifard, Behnam Rahimi and Bijan Esmaeilnejad contributed to conception, design, data collection, statistical analysis and drafting of the manuscript. Behnam Rahimi, Farnaz Malekifard and Bijan Esmaeilnejad contributed to conception, design, supervision of the study and drafting of the manuscript. All authors approved the final version for submission.

## CONFLICT OF INTEREST STATEMENT

None of the authors have any conflicts of interest to declare.

## ETHICS STATEMENT

Ethical Considerations the study was approved by Animal Ethics Committee in Urmia University, Urmia, Iran (IR‐UU‐AEC‐3/36) and conducted under the regulations of this committee.

## Data Availability

The data that support the findings of this study are available on request from the corresponding author. The data are not publicly available due to privacy or ethical restrictions.
